# Implementing large-scale health system strengthening interventions: experience from the better health outcomes through mentoring and assessments (BHOMA) project in Zambia

**DOI:** 10.1186/s12913-018-3619-3

**Published:** 2018-10-19

**Authors:** Wilbroad Mutale, Susan Cleary, Jill Olivier, Roma Chilengi, Lucy Gilson

**Affiliations:** 1 University of Zambia School of Medicine, Box 50110, Lusaka, Zambia; 2 University of Cape Town, School of Public Health and Family Medicine, Cape Town, South Africa; 3 Centre for Infections Disease Research in Zambia, Box 34681, Lusaka, Zambia

**Keywords:** Health system, Implementation, Zambia, Information system

## Abstract

**Background:**

Under the Doris Duke Charitable Foundation’s *African Health Initiative*, five Population Health Implementation and Training partnerships were established as long-term health system strengthening projects in five Sub-Saharan Countries. In Zambia, the Centre for Infectious Disease Research in Zambia began to implement the Better Health Outcomes through Mentorship and Assessments (BHOMA) in 2009. This was a combined community and health systems project involving 42 public facilities and their catchment populations. The impact of this intervention is reported elsewhere, but less attention has been paid to evaluation approaches that generate an understanding of the forces shaping the intervention. This paper is focused on understanding the implementation practices of the BHOMA intervention in Zambia.

**Methods:**

Qualitative approaches were employed to understand and explain health systems intervention implementation practices between 2014 and 2016. We purposively sampled six clinics out of the 42 that participated in the BHOMA project within three districts of Lusaka province in Zambia. At the facility-level we targeted health centre in-charges, health workers, and community health workers. In-depth interviews (*n* = 22), focus group discussions (*n* = 3) and observations were also collected and synthesised.

**Results:**

The major health system challenges addressed by the BHOMA project included poor infrastructure, lack of human resources, poor service delivery, long distances to health centres and inadequate health information systems. In order to implement this in the districts it was necessary to engage with the Ministry of Health and district managers, however, these partners were not actively engaged in intervention design There was great variation in perceptions about the BHOMA interventions. The implementation team considered BHOMA as a ‘proof of concept pilot project’, running parallel to the public health system, while district health officials from the Ministry of health understood it as a ‘long term partner’ and were therefore resistant to the short-term nature of the intervention.

**Conclusions:**

The Normalization Process Theory provided a useful framework to understand and explain implementation processes for the BHOMA intervention in Zambia. We clearly demonstrated the applicability of all the four main components of the NPT: coherence (or sense-making); cognitive participation (or engagement); collective action and reflexive monitoring. We demonstrated how complex and dynamic the intervention played out among different actors and how implementation was affected by difference in appreciation and interpretation of the goal of the intervention. Our findings support the growing demand for process evaluations to use theory based approaches to examine how context interact with local interventions to affect outcomes intended or not.

**Trial registration:**

ClinicalTrials.gov Identifier: NCT01942278. Registered: September 13, 2013 (Retrospectively registered).

## Background

In 2009, the Doris Duke Charitable Foundation (DDCF) supported large-scale and long-term health system strengthening projects in five African countries (Ghana, Mozambique, Rwanda, Tanzania and Zambia). In each country, a health system strengthening intervention was developed and implemented by an external resource team working in collaboration with other actors, usually with a primary health care or district health system focus. The specific focus of each intervention varied, as did the ways in which the interventions engaged with local health system actors [1, 2].

In Zambia, the Centre for Infectious Disease Research in Zambia (CIDRZ) implemented the project called *Better Health Outcomes through Mentorship and Assessments* (BHOMA). It was a combined community and health systems project involving 42 facilities and their catchment populations in rural Kafue, Chongwe and Luangwa districts. BHOMA introduced district-based Quality Improvement (QI) agents, protocolised outpatient care in facilities and established networks of grassroots community presence with teams of Community Health Workers (CHW), Traditional Birth Attendants (TBA) and Clinic Support Workers. Through these health system strengthening and community engagement activities, the BHOMA project intended to reduce the overall age-standardized mortality in these three rural districts [2]. The impact of the intervention (in relation to key health indicators such as adult and under-5 mortality rates) has been reported elsewhere [2–4]. However, much less attention has been paid to the implementation of the intervention, with consideration of the forces that explain the level and pattern of consequences achieved. This paper is focused on understanding the implementation practices of the BHOMA intervention and the potential influences over intervention consequences in Zambia.

### Background: The BHOMA intervention

The BHOMA intervention falls into the category of what is termed a ‘dynamic complex health system strengthening intervention’ as it involved interaction across different domains a local health system [5]. In brief, the BHOMA intervention consisted simple algorithm with protocols which were used by clinicians for diagnosis and management of common diseases seen in most rural primary care settings. The forms for the protocols include the following: patient registration form both for adults and children, antenatal and postnatal forms. These forms were introduced at the health facilities through an onsite intensive training of local staff supported by on site mentorship. The training targeted all members of the facility that included clinicians, nurses and Community health workers (CHWs).

All health workers at each facility were provide with essential diagnostic and management tools to support quality service delivery. The BHOMA project provided limited logistic and financial support for essential supplies and equipment. Monitoring of patient care was done using an electronic medical record system, which reported both quality services offered and patient outcomes in real time. The project also introduced a new cadre called ‘clinic support workers’. This cadre supported the medical record system. At community level, the BHOMA project recruited over 200 CHWs. These were trained and given mobile phones for tracking services in the community. The phones were synchronised with the clinic record system so that data was updated in real time [2, 6].

**Fig. 1:**
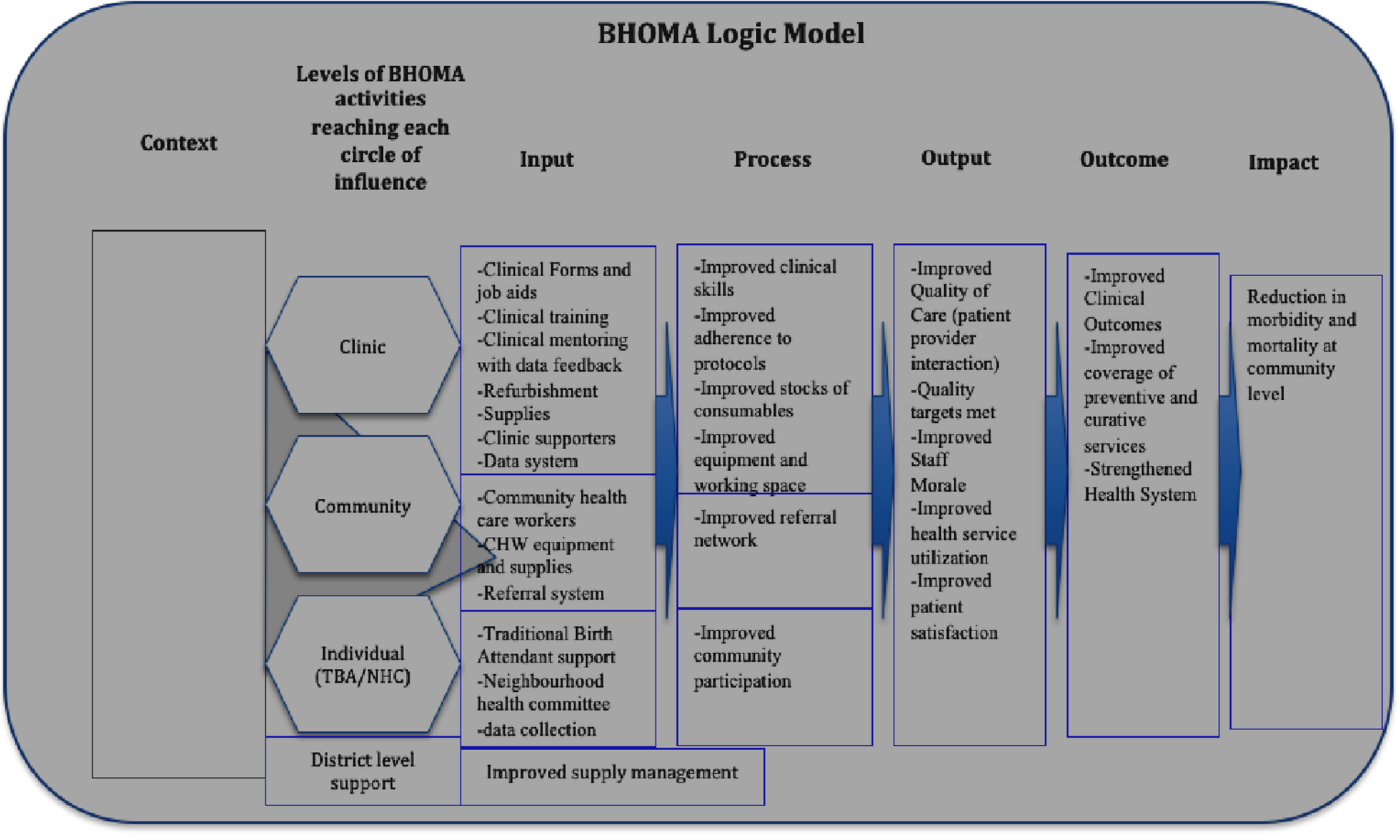
BHOMA logic model. Shows how the original theory of change and result chain for the BHOMA intervention

Figure 1 is the logic model of the BHOMA intervention (from its original protocol documents); it summarises key components of the intervention and the key actors involved in intervention implementation. It highlights the intervention’s intended pathways of change and explores the changes sought by the intervention. This conceptual framework was developed at the time of intervention design (by the intervention’s Principal Investigator and BHOMA core team) and guided the choice of intervention elements. The focus of this paper is the first part of the model (context, input, process implementation). Outcomes are reported in elsewhere [3, 6, 7].

It is understood that complex interventions such as BHOMA are susceptible to the influence of different contextual elements; and may generate unintended positive or negative consequences [6]. The original logic (model) of the intervention might be disrupted – and the impact of such interventions is also strongly influenced by the agents, process and context of implementation [5, 8]. To assess the experience of such interventions in ways that generate knowledge for others involved in health system strengthening, it is, therefore, as important to understand the practices, processes and context of implementation as it is to assess what impact was achieved [6]. Understanding implementation experience can support consideration of how and why an intervention is likely to have generated the impacts observed, and so assist in thinking through the potential for the intervention in other settings. It may also contribute to consideration of how to manage implementation over time and in ways that are likely to support the sustainability of initial goals. This study therefore aimed to document the implementation processes and the context in which the BHOMA study was conducted and how these could impact intended outcomes for the intervention.

## Methods

This article reports on a study conducted by researchers from the University of Zambia and the University of Cape Town (2014–2016), alongside and toward the end of the overall project. This research was understood as an additional layer of reflective research (conducted by researchers external to the intervention team), which was initiated in order to complement the existing research and evaluation activities already being undertaken within the BHOMA project (see above).

Qualitative approaches were employed in this evaluative study, and followed usual HPSR practice [9]. This included an active process of questioning and checking in inquiry (asking how and why things happened and not only what happened, checking answers to questions to identify further issues that needed to be followed up to deepen understanding of the experience); a constant process of conceptualising and reconceptualising (using ideas and theory to develop an initial conceptualisation of the problem or situation of focus and guide data collection, but using the data collected to challenge those ideas and assumptions, and so, being prepared to change ideas in response to the evidence); crafted, interpretative judgements (based on enough evidence, particularly about context, to justify the conclusions drawn, as well as deliberate consideration of contradictory evidence and review of initial interpretations by respondents, such as member checking); and researcher reflexivity (being explicit about how researcher assumptions may influence your interpretation, and testing them in analysis) [10, 11].

The first round of fieldwork and data collection (for this additional research) was undertaken in April 2014, a second round in June 2015, with follow-up in 2016. Information was obtained through in-depth, semi-structured interviews (*n* = 22) and focus group discussions (*n* = 4) with actors who were involved in the implementation of the intervention. Key informants included provincial government officials, members of the BHOMA project and its quality improvement teams, members of the District Health Management Team, health workers in facilities, support workers in facilities, different types of lay workers such as community health workers and traditional birth attendants, and members of community health committees. Observations of key aspects of intervention implementation such as patient flow, data systems, patient consultations, filing systems and data entry was done. For the purposes of this data collection, the intervention was seen as a unique experience of seeking to support health system change. Within this parameter, we used purposive sampling to select the settings (the clinics or districts) in which the data collection took place – as well as the key informants (seeking a range of perspectives and implementation experiences). We worked in three districts and visited six selected clinics. We selected all the districts that took part in the study and in each district we purposefully selected two health facilities, one urban and one rural. We targeted district health managers, health facilities, BHOMA programme managers and implementers. For the FGD’s. we spoke to groups of community health workers who worked with the BHOMA project.

With respect to enrolment, we used standard ethical practices. One of the investigators approached potential respondents with an information sheet, and solicited voluntary written consent once the participant agreed to take part. Notes of interviews, interview transcripts and recordings of interviews that are in an electronic format were only available to personnel involved in the study. These were stored using access privileges and passwords, and given file names in the form of codes (not the names of the respondents). Paper-based records such as printed interviews notes or transcripts were kept in a secure location and accessible to personnel involved in the study. Personal identifiers were removed from research-related information. Confidentiality was maintained during data collection and publication.

Over time, we systematically interrogated and triangulated the evidence of actual experience generated through data collection, to support the credibility of the analysis and the generation of conclusions from it, with these conclusions representing analytic generalisations that are put forward as of relevance and significance for other settings. Three researchers involved in the study coded data manually. Any disagreements were resolved through consensus among the three coders after reviewing the data together. The data was analysed thematically using both directed and conventional content analysis.

We then used Normalisation Process Theory (NPT) as a conceptual framework to interpret the factors which were identified as facilitating or hindering the BHOMA intervention in the three target districts. NPT is concerned with the social organisation of the work (implementation) of making practices routine elements of everyday life (embedding) and of sustaining embedded practices in their social contexts (integration). It presents one approach to enabling an understanding of complex health interventions. It has been used to evaluate e-Health implementation, embedding and integration in high-income countries but rarely has been applied in low-income settings [12–14].

NPT aims to explain the routine embedding of practices by reference to the role of four generative mechanisms: *coherence; cognitive participation; collective action and reflexive monitoring*.

**Coherence:** refers to the work of making a complex intervention hold together and cohere to its context, how people ‘make sense’ or not of the new ways of working (for example, BHOMA stakeholders perspectives and sense-making of the BHOMA intervention).

**Cognitive participation:** is the work of engaging and legitimizing a complex intervention, exploring whether participants buy into and/or sustain the intervention (for example, whether stakeholders ‘bought into’ the BHOMA intervention).

**Collective action:** examines how innovations help or hinder professionals in performing various aspects of their work, issues of resource allocation, infrastructure and policy, how workload and training needs are affected and how the new practices affect confidence in the safety or security of new ways of working.

**Reflexive monitoring:** is the work of understanding and evaluating a complex intervention in practice, and how individuals or groups come to decide whether the new ways of working are worth sustaining.

We applied all of these to the BHOMA intervention (see discussion below).

## Results

The BHOMA intervention was designed to address health system challenges in improving quality of care. Based on the formative work and Ministry of Health reports, the major health system challenges included poor infrastructure, lack of human resource, poor service delivery, long distance to health centres and poor health information.

*“The first problem was the structure (the systems architecture). The second problem was human resource – it doesn’t just need to be doubled, it needs to be quadrupled…we have one of the highest population growth stats here, so the problem is only going to get worse. The third problem is infrastructure – which is a much worse problem than the others – a lot of the health facility buildings need work. The government is close to their goal of a facility within 5km of all – but trained staff does probably not man those facilities. The HMIS, it is not accurate, very behind – [you] can’t see the trends at all (it takes over 4 months for data just to be entered)”. R 1 (IDI)*

This conceptualisation of BHOMA was formed out of the experience and history of the BHOMA Principal Investigators (PIs) who had worked in quality improvement in the context of HIV services in Zambia. They pioneered the use of CHWs and task-shifting, and introduced protocols for HIV patient care in Zambia. Based on this success, with BHOMA they sought to replicate this on a larger scale – as a health system improvement, but based on their HIV experience.

*“…BHOMA was designed on the basis of what PIs wanted to do with ARVs/HIV care which was based on the quality improvement (QI) cycle…there is a tendency to apply QI concepts to everything, so that is how the same thinking was extended to the BHOMA intervention” R1 (IDI)*

However, in order to implement this in targeted districts, it was necessary to engage with the Ministry of Health and district managers. However, initial engagement was mainly focused on gaining buy-in rather than designing the intervention together.

“*In Zambia you can’t do anything at a health facilities without the approval of permanent secretary and district director. BHOMA had to be endorsed – but for some reason, the levels of appreciation are not good – when asked they say, ‘oh I’ve heard about that’…the relationship with the district is very important – we had a series of meetings and told them about the objectives and outcomes of the BHOMA project – the relationship is quite good with them but appreciation of the project is low.” R3 (IDI)*

During the BHOMA intervention period (2009 to 2015), there were several major changes to the local health system that impacted on implementation, and on this relationship with the public sector. For example, as the intervention evolved, there were several changes in senior personnel in the Ministry of Health; the BHOMA intervention districts were reconfigured (during the intervention they were redrawn, from 3 to 5 districts); and the Ministry of Health was split into two separate Ministries. This made it difficult to coordinate activities and relationships sustainably through the intervention.

“*We worked with three people in the MOH at first – two moved away, and the other one is very busy … we have been trying to get our slot (at national level MOH) to tell them about BHOMA but we are still waiting.” R1 (IDI)*

### Making sense of the BHOMA implementation

A key finding of this research was that there were highly varied interpretations of the BHOMA intervention’s purpose and character among stakeholders. However, these interpretations can be divided into two broad camps, based on whether stakeholders understood the BHOMA intervention to be a *research study* or a *health systems strengthening (HSS) intervention*.

At the central level (for example, at the CIDRZ head office in Lusaka, and among the BHOMA design team), most staff understood BHOMA to primarily be a *research study* with a limited time-frame (so the activities were piloted, and the main focus was on ‘proof of concept’). This was in contrast to district health officials and others from the Ministry of Health who understood the BHOMA intervention to be a long-term HSS investment, and were therefore less prepared to accept the short-term nature of the intervention.

*“…the community thinks we are there for long term service delivery.” R12 (FDG)*

*“We called the DMOs [District Medical Officers] and told them we would be leaving over 2 or 3 months…they were not happy” R2 (IDI)*

We explored what different actors considered as important components of the BHOMA intervention. All respondents acknowledged that the BHOMA intervention was a complex intervention, consisting of several interconnected components.

“*BHOMA looks at PHC as a whole. Others just look at HIV or TB. That’s the biggest difference. Also support with staffing and equipment is a big difference. TB organizations just provide TB support. BHOMA attends to all problems[whole system].” R1 (IDI)*

Respondents weighted the importance of different components of the BHOMA intervention differently, depending on the level of engagement with BHOMA. The major components identified by different actors included:

- **Training and mentorship:** QI teams training health workers and providing mentorship.
- **Improved information and feedback:** Use of ‘forms’ which collected data, then CHW inputted into dedicated simple computer WHERE, which were connected to district and central server to use for decision making*.*
- **Use of standards and protocols:** For standardised clinical assessments health workers.
- **Strengthening CHWs/(Clinic suporters):** Were by the project, trained and given a monthly stipend as an allowance.. They were also given hand-held mobile phones for CHWs feeding into new info system, including referral and return reminders.
- **Task-shifting and clinic support staff:** CHWs trained and (stipend) salaried and placed into health centres to assist in doing basic vitals of patients and to do triage and admin (e.g. filing).

It was the combination of components which were seen as unique and important for the BHOMA project. Training and mentorship coupled with motivation through stipends was effective in motivation and retaining CHW who in turn supported health workers and clinic activities.

### Human resource mentorship and training

It was revealed that when the BHOMA mentorship team started implementation of the intervention in target clinics, they faced resistance by clinical staff who did not feel the BHOMA staff were (better) qualified to act as mentors. In some cases, mentees left the stations and patients to mentors - opting to attend to other issues instead of sitting together with mentors to see patients as intended. The approach used to overcome this initial resistance was described as a ‘humble approach’ by BHOMA team-members. They first proved themselves useful and earned the trust of mentees overtime and those who were initially sceptical later appreciated the support from the BHOMA team, and were happy to be mentored and supported. Mentors also emphasised that they did not come to the facility ‘as supervisors’ but rather as ‘fellow workers’ who strategically assisted with patients before handling BHOMA specific issues.

*“To be a coach and not a supervisor – this was emphasized during training. A supervisor issues instructions and rebukes, a coach works together through a task and follows-up” . R5 (IDI)*

*“We go and first help with the clinical activities, and then once the centre is quiet, then we mentor – you can’t mentor if they are busy” R8 (IDI)*

The BHOMA mentorship team also used the BHOMA (CIDRZ) vehicle to support clinics and the district office. This was greatly appreciated, especially when transporting supplies to clinics or supporting supervisory visits. The vehicles were also used to transport patients to hospitals in the absence of an ambulance.

*“We have benefited from the BHOMA project vehicle especially during emergencies when our ambulance is out of service which happens now and then” R13 (IDI)*

The vehicle sharing was not overtly part of the BHOMA intervention strategy (the logic model), but instead was a characteristic of the implementation practice. It was these kinds of ‘quasi-in-kind’ support that were most appreciated by the health workers and district-level staff.

According to the intervention design, the BHOMA team was intended to work closely with the District Clinical Care Specialist, who was the main contact person at the district – mainly in relation to site mentorship and supervision. However, this did not always work out in the actual implementation, mainly due to conflicting programmes for the District Health Team, who were usually not available to accompany the BHOMA team for supervisory visits to the study sites.

*“No, it doesn’t always or often work for DOH to go along to the site visits [facility visits or training]…more often than not it is only the QI [BHOMA Quality Improvement] teams that go” R1 (IDI)*

### Implementation of systems ‘hardware’: Mobile phones, stationary, and computer interface

The information system (based on mobile phone technology) was designed to assist CHWs to collect community-level data, which was synchronised with facility data (and loaded to a server at the CIDRZ office in Lusaka). The phones were also used to send reminders for following up severe cases in the community.

However, at the time of main fieldwork (2014 and 2015), when we asked whether CHW mobile phones were working as intended, it was noted that a number of them were not functioning (for example, had broken down and had been sent to the head office for repairs). BHOMA team members assessed that about 40% of the cell phones were still working – although some still with minor challenges. Talking to the programme managers, there was no contingency plan to replace the cells phones. The fact that the project was heading towards completion when this implementation research was conducted, and that it was understood that the MoH would not take over this component - made it less desirable to replace the phones. In terms of impact on the project activity, this resulted in poor community data and follow-up of patients in the community with time. However, more relevant to the focus of this paper, it also impacted on the implementing staff, who described a certain level of despondency and frustration at not being able to ‘fix’ this aspect of the implementation.

“*When we started we bought equipment which has broken and we can't buy more”. R10 (FGD, Luangwa)*

*“Most [phones) broke down some months ago – so now they come in and work at the clinic instead of going out to the zones” R11 (FDG, Chongwe)*

The project also provided stationary throughout the life of the intervention – which was highly appreciated by all staff and CHWs interviewed. Stationary included, for example, forms for patient records and folders, pens, and record books. Interestingly, while the stationary was not a major intervention feature (in the program logic), it ended up being a major focus – and also a significant sustained expense to the project budget. The monthly cost of stationary consumed at facilities ranged from 1,000 Zambian kwacha (≈100 USD) for small sites to 2,500 Zambian kwacha (≈ 250 USD). However, like the mobile phones, the discontinuation (or in some cases the impending discontinuation) of the stationery support caused significant frustration, with facility staff and officials noting concerns about what they would do at the end of the project. The MoH officials indicated that it was not possible to take over this cost as they received far less from the central government to support their operations.

*“The district team would say the programme is very good but it’s very expensive and in my own understanding I've seen that sustainability in most of these projects is a problem” R14 (IDI)*

The capture of data was done on simplified user-friendly touch screen computer terminals – by CHWs employed by the project at the facility-level. Generally all the computers were functional and connected to the central server. Although data entry was meant to be in real time, it was not always the case as there were recorded delays to complete the data entry in many places for reasons such as workload, faults in the operating system and cell phone breakdowns. It was also observed that while BHOMA data were available at the health centre and districts offices for decision-making, it was hardly used. The health facility In-charges were given access privileges to the electronic data, but hardly ever used the data for their day-to-day management decision. This situation was the same at district level, where only District Health Information Officers were fully aware of the BHOMA data; perhaps because of the expectation on them to review the data and share with their managers.

It was acknowledged that the same data was collected twice – both for the project and for the government HMIS system resulting in duplication of work.

*“We have a challenge. In sites where staff accepted the programme it was easy to take them through but if there was resistance to change we had a challenge. We would show the in-charge how to look at data but at other sites it was a challenge. You show them how to view and you go back the next month and find they have not even viewed the indicators.” R13 (IDI)*

Discussions with the BHOMA team at the district level indicated that while the data were available at the clinic, the emphasis had been on using the data to support mentorship. It was evident from the responses that the major use of the data was not necessarily for decision-making at health facility or district level.

*“We have very little evidence on that, very little action. The reason they (DHO) don’t use it [the data], they are not bad guys, they are captaining very turbulent waters, and get tossed around a lot… they lack of funding and HR, and if a politician comes past they have to drop everything – they probably spend 90% of their time attending to such visitors.” R1 (IDI)*

*“They don’t care [about information]. Do DHOs have any use for this information? We keep explaining this – we are not doing well in that area.” R7 (IDI)*

### Role and impact of on CHWs

The BHOMA CHWs were selected from the local communities where the intervention was being implemented. They had three main responsibilities: patient registration, triaging and following up patients in the community. All key stakeholders appreciated the presence of CHWs as they helped to reduce the workload for clinicians. They also helped to collect community-level data and thus helped to follow-up complicated cases.

*“CHWs are very effective support for clinic staff – giving more time to interact with patients.” R5 (IDI).*

CHWs were very motivated and were supervised by health centre In-charges with minimal oversight from BHOMA team. This made this cadre very effective and accountable. They made patient feel comfortable and welcome. They also picked up serious cases for faster consultations (triage) while keeping records and files in order. In the afternoon they spent time entering the data from files into the electronic medical record system. Other CHWs were given patients to follow-up via the project cell phones. Once the case outcome was determined it was entered into the cell phone software which synchronised with facility data upon connection to mobile network. The CHW received a monthly stipend equivalent to $60 which was said to be a major incentive for them.

It was clear that while the CHWs were generally appreciated by those in the system, this appreciation was not enough to be able to leverage (limited) local resources for the continuation of CHWs beyond the BHOMA study period. Given the lack of engagement at the national level (described above), there was no discussion on possibilities to integrate BHOMA CHWs into the health system (for example, if the MoH could take over paying stipends). Interestingly, the MoH introduced CHW training and began employing them, so there was an opportunity available for engagement on lessons learnt from the BHOMA implementation.

### Relationships and ownership

The relationship between the District Health Management Team (DHMT) and the BHOMA team was reported to be excellent, and was noted to have led to mutual trust. The reputation of the DHO had improved because of the contribution from BHOMA activities, and because the credit for this was ascribed to the DHO (see below on ownership). However, this also added stress, as there was therefore a lot at stake should BHOMA pull out because the officials were aware that the evident ‘improved service’ might come to an end (and other tangible differences, such as patients needing to purchase their own notebooks as health records without the stationary supply). The fact that this was a limited time-framed intervention, therefore put strain on the relationship between the project and health officials.

The BHOMA QI team needed to accommodate the DHO who had several other commitments. This meant re-scheduling meetings or supporting important district activities while waiting for an opportunity to engage with DHO.

*“There has to be a high level of flexibility (laughs)…All stakeholders – a lot of give and take before we get to the improved quality we are aiming for. Really close networking is required with the DMOs – have to tell them at every step – have to negotiate each step and approach them properly.” R2 (IDI)*

It was reported most respondents that the BHOMA intervention worked better in districts or clinics where the local team engaged with the project. Where the facility manager was motivated and engaged closely with the intervention, the activities were smooth and more likely to be accepted.

*“We are learning – the sites where the in-charge took ownership from the start, that works well – especially the BHOMA forms and some health workers feel they take too much time – several resisted … staff took ownership and accepted it’s good for them and the community.” R3 (IDI)*

### Challenges during implementation

High staff turnover was a major challenge across all study sites, and the problem affected all levels of the intervention. For example, health workers were regularly transferred without prior notice, which meant that new staff had to be re-trained and mentored. CHW attrition was also very high, thus affecting the intervention implementation in some sites. In addition, the BHOMA implementation team also changed several times.

*“There is a massive level of attrition in districts, HR turnover, new personnel in facilities, had to put in a lot of effort…we just keep training – the attrition is out of control” R2 (IDI)*

*“The turnover of lay staff – a huge challenge – so we have to train and train and train again – and then the performance indicators go down… we expected this, but not to this extent” R1 (IDI)*

There were also challenges with the cell phone network and electricity supply. In two remote sites, costly satellite dish procurement and installation was required to enable access to the internet. In many more places (at least 15 sites) solar panel and battery installations were needed to provide electricity supply to the clinic.

*“ Challenges are network connectivity in the community. We had to setup hotspots in clinics; also no electricity so can’t charge phones – so gave them solar mobile phone chargers that costed USD50 each – we bought about 100 of these.” R4 (IDI)*

Added to the staff turnover challenge already described, this demonstrates how sustainability of systems ‘hardware’ was a major challenge that inhibited the implementation *during* the intervention.

Some aspects of the intervention were judged to be more worthy of continuation after the end of the BHOMA project (in a low-resourced environment) than others. Although there was variation in preference, at a facility-level, the forms (entry-registration and assessment forms) where seen as an important innovation; and also maintaining CHWs. However, as noted earlier, while the DHO also showed appreciation for these, it was noted that there were no resources to allocate to this (or take ownership of it) from central government.

*“One facility came and said we loved the protocol forms, please could we have a 3 month supply, so we did. [The equipment]…the DMO could keep it – but there would no longer be a data panel there to support the data system, so the data system is now gone from 6 sites pilot sites…. the volunteers are gone, and there are no data capturers anymore.” R3 (IDI)*

## Discussion

We applied the four Normalisation Process Theory constructs in order to assess these findings further.

### Coherence

Coherence refers to the ‘sense-making’ work undertaken when a new intervention is implemented. This is to determine whether users see it as differing from existing practice, have a shared view of its purpose, understand how it will affect them personally and grasp its potential benefits [13, 15]. It was clear that considerable effort was put in designing and implementing the BHOMA intervention. There was evidence to suggest stakeholders knew about the existence of the BHOMA intervention, and the core activities. In terms of what the goals of the project were, it was agreed that the intervention was very big and addressing a complex problem. However there were different levels of appreciation towards the nature of intervention. For the designers and lead implementers of the BHOMA intervention, it was a trial research project with very clear timelines. In contrast the MoH officers and the community members interpreted BHOMA as a more sustainable (longer-term) health programme that was there to help them run primary health care. The health system stakeholders did not understand that an intention was for the system to ‘host’ activities as a way to sustain the programme after the project end. It was therefore unsurprising that the district health team and the community members did not feel prepared for the ending of the BHOMA intervention, and did not have a contingency plan in place to continue the valued activities.

### Cognitive participation

Cognitive participation focuses upon the work undertaken to engage with stakeholders and beneficiaries and get them to ‘buy into’ a new intervention [13, 15]. This is crucial to the successful implementation of any new technology. A protocol-driven intervention such as the BHOMA intervention required the MoH headquarters, DHO, health facility managers, health workers and the community to understand the purpose of the intervention and engage with it according to their role in the health system. The MoH understood the need for health system strengthening and have articulated this in their 2011–2015 action plan. It was therefore not difficult to accept the introduction of the BHOMA intervention in the target districts. The districts were very engaged in the design and played a pivotal role in deciding the elements and the implementation of the project – and it can be said that the District Clinical Directors were ‘embedded’ into the BHOMA implementation team. The BHOMA implementation team was employed directly from the project and had separate office space and transport. This was a point of weakness that made it difficult to integrate the BHOMA into the district health team. They were seen as a separate but supportive donor-driven project.

At the health facility level, the initial interaction with the In-charge was central to accepting the intervention. Initially there was resistance for the mentee to accept the mentors from the project. This was so because the mentors were fellow clinical officers and nurses. Apart from being oriented under the project they did not possess any extra qualifications. In fact, in some cases the mentors were left to see patients alone. Working with the district office, the health workers finally accepted to be mentored. It required ongoing follow-up and trust in order for the process to work. Challenges faced included transfer of staff and the reporting structures for uncooperative health workers. The BHOMA team was not given the power to discipline HW but had to go through the district director’s office.

### Collective action

The emphasis of collective action involves the work performed by individuals, groups of professionals or organisations in operationalizing a new technology or intervention [13, 15]. In the BHOMA intervention, most of the stakeholders accepted the need for having standardized protocol-care but it was clear that the design of the intervention emanated from the central and districts levels with less input from the clinical staff and the community. This can explain the observed non-use of some key elements of the intervention such as electronic medical records for decision-making. The health workers accepted elements that appeared much more useful for them such as clinical support workers who helped to reduce their workload. Community also appreciated the intervention for different reasons such as the availability of free medical files and having vital signs checked and being seen faster than before. If, at design stage, all the stakeholders were involved and appreciated the elements included the intervention may have worked better [8]. Such participatory approaches would have helped to maximize the benefit that the BHOMA intervention intended to achieve.

### Reflexive monitoring

Reflexive monitoring deals with the evaluation and monitoring of intervention implementation and how this is used to improve ongoing/future implementation [13, 15]. Much of the reflexive monitoring in the BHOMA intervention was to support the mentorship process. The BHOMA central office and district team paid attention to performance indicators, which they used to isolate health workers and clinics that needed further support. This was done in real time. However, the HF and District health office rarely looked at performance indicator for decision-making or supervision. CHW also never used the data to understand their own performance. The EMR was therefore under utilized for local decision-making.

Literature has shown that Normalisation theory can help understand important drives for promoting better performance of complex interventions [16–18]. In this study, we used NPT to explain how the BHOMA intervention worked, looking at both early implementation and, considering how it became embedded into routine practice in Zambia. These lessons are applicable to evaluation of complex interventions in similar settings.

In our study, we focused on what individuals and groups contributed to allow for the normalisation of the intervention. Clearly, we demonstrated the applicability of all the four main components of the NPT which are: coherence (or sense-making); cognitive participation (or engagement); collective action and reflexive monitoring [18]. We demonstrated how complex and dynamic these components are and how together affected implementation of the BHOMA project positively or negatively [13, 15, 16].

It is understood that the overall goal of doing research into complex interventions is to better understand drivers of good performance [16, 19]. This calls for researchers to develop interventions that can be adopted and implemented in different settings [16, 19]. Unfortunately, several trials fail to explain the processes that were critical to intervention acceptability and embeddedness [19–21]. This can explain the observed ‘know-do’ gap between evidence about effective interventions and their transfer into clinical practice, as implementation information is key to replication and adaptation [16].

#### Study limitations

The main strengths of this study lie in fact that we have used health systems research approach to evaluate the process implementation of complex health intervention looking at actors and context and how these interacted with the intervention. In addition we applied a robust analytical framework, combining information generated with and implementation process theory (NPT) in order to analyse actors perspectives on the BHOMA intervention. The study had a number of limitations – and given that it was qualitative in nature, generalization of the findings must be done with caution. In addition, some participants from the implementation team could have under-reported the negative consequences of the study (or over-reported the appreciation or effects of the implementation). All efforts were made to ensure the stakeholders understood that this was not an evaluation study (that is, not evaluating impact, and therefore not influencing future funding), but given the particular tensions in play in this context (with the project ending, and activities under threat), it is possible that this still influenced the findings.

## Conclusions

The Normalization Process Theory provided a useful framework to understand and explain implementation processes for the BHOMA intervention in Zambia. We clearly demonstrated the applicability of all the four main components of the NPT: coherence (or sense-making); cognitive participation (or engagement); collective action and reflexive monitoring. We demonstrated how complex and dynamic the intervention played out among different actors and how implementation was affected by difference in appreciation and interpretation of the goal of the intervention. Our findings support the growing demand for process evaluations to use theory based approaches to examine how context interact with local interventions to affect outcomes intended or not.

## Abbreviations

BHOMA: Better Health outcome Through Mentorship and Assessment
CIDRZ: Centre for Infectious Disease Research in Zambia
DDCF: Doris Duke Charitable Foundation
NPT: Normalisation Process Theory
QI: Quality Improvement
